# Radiofrequency Enhancer to Recover Signal Dropouts in 7 Tesla Diffusion MRI

**DOI:** 10.3390/s24216981

**Published:** 2024-10-30

**Authors:** Varun Subramaniam, Andrew Frankini, Ameen Al Qadi, Mackenzie T. Herb, Gaurav Verma, Bradley N. Delman, Priti Balchandani, Akbar Alipour

**Affiliations:** 1 Department of Diagnostic, Molecular and Interventional Radiology, BioMedical Engineering and Imaging Institute (BMEII), Icahn School of Medicine at Mount Sinai, New York, NY 10029, USA; varun.subramaniam@icahn.mssm.edu (V.S.);; 2 Nash Family Department of Neuroscience, Icahn School of Medicine at Mount Sinai, New York, NY 10029, USA

**Keywords:** RF resonator, diffusion MRI, inductive coupling, ultra-high field MRI

## Abstract

Diffusion magnetic resonance imaging (dMRI) allows for a non-invasive visualization and quantitative assessment of white matter architecture in the brain by characterizing restrictions on the random motion of water molecules. Ultra-high field MRI scanners, such as those operating at 7 Tesla (7T) or higher, can boost the signal-to-noise ratio (SNR) to improve dMRI compared with what is attainable at conventional field strengths such as 3T or 1.5T. However, wavelength effects at 7T cause reduced transmit magnetic field efficiency in the human brain, mainly in the posterior fossa, manifesting as signal dropouts in this region. Recently, we reported a simple approach of using a wireless radiofrequency (RF) surface array to improve transmit efficiency and signal sensitivity at 7T. In this study, we demonstrate the feasibility and effectiveness of the RF enhancer in improving in vivo dMRI at 7T. The electromagnetic simulation results demonstrated a 2.1-fold increase in transmit efficiency with the use of the RF enhancer. The experimental results similarly showed a 1.9-fold improvement in transmit efficiency and a 1.4-fold increase in normalized SNR. These improvements effectively mitigated signal dropouts in regions with inherently lower SNR, such as the cerebellum, resulting in a better depiction of principal fiber orientations and an enhanced visualization of extended tracts.

## 1. Introduction

Diffusion magnetic resonance imaging (dMRI) is an MRI technique that assesses water molecular diffusion in biological tissues. dMRI provides insight into the microscopic details of the tissue microstructure as molecules diffuse variably throughout tissues based on the local microstructure [1,2]. Water molecules preferentially diffuse parallel to the longitudinal axis of white matter axons and are restricted perpendicular to these axons, resulting in diffusion directionality called anisotropy [3,4]. The information obtained from dMRI can be utilized to assess additional tissue characteristics, such as fractional anisotropy (FA), mean diffusivity, and connectivity. dMRI has been widely used in investigating various brain pathologies, including epilepsy, stroke, and other neurological disorders [5,6,7,8].

Ultra-high-field (UHF) MRI at 7 Tesla (7T) or higher field strengths improves the signal-to-noise ratio (SNR) compared to lower field strengths such as 3T and 1.5T [9,10,11,12,13]. The increased SNR translates to the improved visualization of anatomical structures, thus enhancing its diagnostic utility. Despite these benefits, the shorter wavelength associated with the increased frequency at 7T causes inhomogeneity in the transmit field ($B_{1}^{+}$) when using the conventional single-channel transmit (1Tx) head coils. This leads to flip angle non-uniformity over the brain [14,15]. Typically, a 1Tx head coil is driven in quadrature mode generating circularly polarized (CP) excitation with highly homogenous $B_{1}^{+}$ at lower field strengths (1.5T and 3T). However, at 7T, CP excitation confers inhomogeneous $B_{1}^{+}$ in the human brain, with lower transmit efficiency especially in the posterior fossa, which results in signal dropouts in this challenging region. Additionally, in diffusion sequences, where a uniformly high-energy refocusing pulse is strongly preferred, the increased RF energy leads to restrictions due to specific absorption rate (SAR) limits. Therefore, with $B_{1}^{+}$ inhomogeneity increasing with field strength, developing techniques to mitigate such inhomogeneity becomes important, particularly at UHF.

This inhomogeneity can be reduced using active RF shimming, RF pulse design, and passive RF shimming techniques. For example, parallel transmit (pTx) is an active RF shimming method that uses multiple independently driven RF transmission channels to improve $B_{1}^{+}$ homogeneity by controlling the magnitude and phase of each channel [16,17]. The use of the pTx method for human connectome project on whole-brain dMRI at 7T showed major advantages in the rapid acquisition of high-quality data [13]. However, the requirement of multiple channels and RF amplifiers make this technique particularly complex. Another approach to reduce $B_{1}^{+}$ inhomogeneity is to use adiabatic pulses that generate a quadratic phase distribution to allow the phase to be fully refocused [18]. Although adiabatic RF pulses can deal with $B_{1}^{+}$ inhomogeneity issues, they are typically associated with a high RF power deposition, meaning that SAR restrictions at UHF become an even bigger problem. Passive RF shimming techniques such as the placement of dielectric pads represent simple hardware solutions to alter the $B_{1}^{+}$ field distribution and increase the $B_{1}^{+}$ homogeneity in the most affected brain regions [19,20,21,22]. Such dielectric pads have improved the 7T dMRI in the regions with lower $B_{1}^{+}$, such as the cerebellum and temporal lobe. Dielectric pads are small bags filled with a suspension of high-permittivity material that can be placed inside the head coil to be close to brain, but dielectric pads may lose their electrical and physical properties over time. Various other passive RF shimming approaches, such as metamaterials, metasurfaces, and meta-arrays, have been documented in various studies to mitigate the wavelength effect in UHF systems [23,24,25,26,27,28].

In our recent study, we reported a reliable wireless RF enhancer that was based on an inductive coupling between the RF enhancer and the CP 1Tx head coil to improve the transmit efficiency while avoiding the hardware complexity and other disadvantages of these prior methods [29,30]. Here, we demonstrate the advantages of using the RF enhancer in improving in vivo whole brain dMRI at 7T.

## 2. Materials and Methods

### 2.1. Theoretical Background

**Figure 1:**
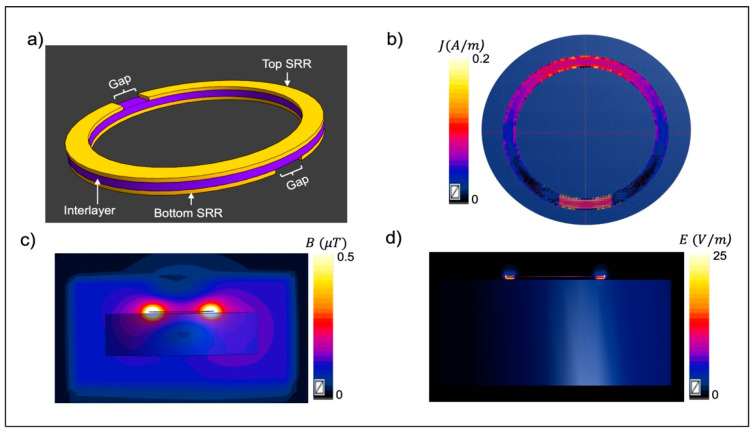
(**a**) Three-dimensional schematic of a BC-SRR. (**b**) Surface current distribution along the rings. (**c**) Cross-section view of the magnetic (H) field distribution generated by the BC-SRR. (**d**) Cross-section view of the electric (E) field distribution generated by the BC-SRR.

Considering a single wireless RLC resonator inside an MRI coil, interaction of the electromagnetic (EM) field generated by the MRI coil with the wireless resonator through inductive coupling results in enhancing the magnetic (H) and electric (E) fields in the vicinity of the resonator [31,32,33]. The H-field enhancement can be used to improve MRI coil $B_{1}^{+}$ efficiency in the regions with intrinsically lower efficiency. Nevertheless, the enhancement of the E-field, particularly near the capacitor, may result in elevated SAR, raising safety concerns related to tissue heating. This issue might be addressed by replacing the conventional RLC resonators with multilayer printed circuit resonators technology to confine E-field generated by the resonator without allowing to distribute [32]. In our previous works, we have studied various multilayer resonator architectures to use in RF sensing [28,29,30,31,32,34,35]. One design that is well known from the microwave/metamaterial community is the broadside-coupled split-ring resonator (BC-SRR). The BC-SRR is composed of two conductive resonators and a dielectric substrate (interlayer) sandwiched between the top and bottom rings [36,37]. Each ring has one gap, and the rings are placed across the interlayer such that the gaps are aligned counter-oriented (Figure 1a). The EM field applied externally by the MRI coil excites the BC-SRR inducing a current on the resonator (Figure 1b) and consequently results in a secondary H-field (Figure 1c). The associated E-field is built along the capacitive region and across the gap due to the charge stored across the gaps and the interlayer (Figure 1d). These stored H-fields and E-fields lead to a resonant spectral response and form a RF resonator.

The BC-SRR structure can be modeled by a simple series RLC resonator circuit with a single lumped capacitance and lumped inductance, where the per unit capacitance ($C_{u})$ and inductance ($L_{u}$) are given by Equations (1) and (2), respectively. The resonance frequency ($f_{0})$ of the resonator can be calculated using Equation (3) [38].

(1) $$C_{u}=\frac{1}{2}\varepsilon_{0}\varepsilon_{eff}\left(\frac{2w_{d}}{h}+1.393+1.667+0.667\ln\left(\frac{2\omega_{d}}{h}+1.444\right)\right)\;$$

(2) $${\;L}_{u}=0.00508l\left(2.303{log}_{10}\frac{4l}{d}-2.451\right),\;in\;the\;unit\;of\;\mu H$$

(3) $${\;f}_{0}=\frac{1}{2\pi \sqrt{C_{u}L_{u}}}$$

where $\varepsilon_{0}$ is the permittivity of free space, $\varepsilon_{eff}$ is the effective permittivity of the capacitive region between two conductive layers, $w_{d}$ is the metallization width, h is the thickness of the capacitive region, l is the conductor length, and d is the conductor thickness.

In the case of placing BC-SRR inside the MRI coil, interaction of the resonator with the CP magnetic field, $B_{rf}$, generated by the MRI coil leads to circulating current in the resonator, which results in a secondary magnetic field, $B_{re}$, in the resonator vicinity.

Assume the CP magnetic field generated by the MRI coil (quadrature birdcage) expressed as Equation (4) [39,40].

(4) $$B_{rf}\left(t\right)=B_{1}^{+}\left(cos\;\omega_{L}t\;i-sin\;\omega_{L}t\;j\right)$$

$B_{1}^{+}$ is an amplitude modulation function and $\omega_{L}=2\pi f_{L}$ is the carrier frequency of the transmission operating in Larmor frequency.

The inductive coupling between the resonator and $B_{rf}\left(t\right)$ results in a linearly polarized magnetic field ${(B}_{re})$ generated by a resonator, which can be expressed as Equation (5).

(5) $$B_{re}\left(t\right)=2B_{re}^{+}\cos\omega_{L}ti$$

Assume the angle between CP magnetic field lines and normal vector of the resonator is zero; therefore, from Faraday’s law of induction, the electromotive force (ε) generated by $B_{rf}\left(t\right)$ is given:

(6) $$\epsilon =-\frac{d\phi }{dt}=\left(\pi r^{2}\right)\omega_{L}B_{1}^{+}$$

where ϕ is the magnetic flux and r is the radius of the resonator.

If the resonator is considered as a series RLC circuit, the input impedance can be written as

(7) $${\;Z}_{re}=R\left[1+i\frac{\omega_{L}L}{R}\left(\frac{f_{L}^{2}-f_{0}^{2}}{f_{L}^{2}}\right)\right]$$

where R represents the ohmic losses, L is the resonator inductance, and $f_{0}=1/2\pi \sqrt{LC}$ is the resonance frequency of the resonator. Assume Δ$f=\left(f_{L}-f_{0}\right)$, the impedance can be simplified to

(8) $$Z_{re}={-i2\omega }_{L}L\left(\frac{\Delta f}{f_{L}}\right)$$

The associated ohmic loss, R, is typically small; therefore, the induced current on the resonator can be written as

(9) $$I_{re}=\frac{\epsilon }{Z_{re}}≃\frac{\left(\pi r^{2}\right)B_{1}^{+}}{2L}\left(\frac{f_{L}}{\Delta f}\right)$$

The modulation magnetic field generated by the induced current at distance z away from the resonator center is given by

(10) $$B_{re}\left(t\right)≃\;\frac{\mu \left(\pi r\right)B_{1}^{+}}{L{\left(1+{\left(\frac{z}{r}\right)}^{2}\right)}^{\frac{3}{2}}}\left(\frac{f_{L}}{\Delta f}\right)cos\;\omega_{L}ti$$

This linearly polarized field decomposes into two CP magnetic fields [30,41]. One is a circularly forward-polarized field and the other is a circularly reverse-polarized field, which mathematically can be expressed as Equation (11):

(11) $$B_{re}\left(t\right)≃\;\frac{\mu \left(\pi r\right)B_{1}^{+}}{2L{\left(1+{\left(\frac{z}{r}\right)}^{2}\right)}^{\frac{3}{2}}}\left(\frac{f_{L}}{\Delta f}\right)\left[cos\omega_{L}ti-sin\omega_{L}tj\right]+\frac{\mu \left(\pi r\right)B_{1}^{+}\;}{2L{\left(1+{\left(\frac{z}{r}\right)}^{2}\right)}^{\frac{3}{2}}}\left(\frac{f_{L}}{\Delta f}\right)\left[cos\omega_{L}ti+sin\omega_{L}tj\right]$$

Equation (11) represents a linearly polarized magnetic field that consists of two circularly polarized magnetic fields: the forward-polarized magnetic field (the first term) and the reverse-polarized magnetic field (the second term). Since proton spins are only sensitive to the forward-polarized magnetic field, the actual modulation of $B_{1}^{+}$ along the axis of the resonator corresponds only to the first term of Equation (11). Therefore, we neglect the second term, which does not affect spin excitation, and consider only the forward-polarized field. This field is more resonant with the spins and rotates in the same direction as the precessing spins.

Therefore, the total magnetic field at the distance z from the resonator center is explained by Equation (12):

(12) $$\begin{array}{c} B_{t}^{+}\left(t\right)≃\;B_{rf}\left(t\right)+\frac{\mu \pi B_{1}^{+}}{2L{\left(1+{\left(\frac{z}{r}\right)}^{2}\right)}^{\frac{3}{2}}}\left(\frac{f_{L}}{\Delta f}\right)\left[cos\omega_{L}ti-sin\omega_{L}tj\right]\\ \;\;\;\;\;\;\;\;\;\;\;\;=\left[1+\frac{\mu \pi }{2L{\left(1+{\left(\frac{z}{r}\right)}^{2}\right)}^{\frac{3}{2}}}\left(\frac{f_{L}}{\Delta f}\right)\right]\;B_{1}^{+}\left[cos\omega_{L}ti-sin\omega_{L}tj\right] \end{array}$$

$where\;B_{1}^{+}$ is the original magnitude of $B_{t}^{+}\left(t\right)$ when there is no resonator in place. Considering a resonator in this study tuned below the Larmor frequency ($f_{L}>f_{0})$, the total magnetic field, $B_{t}^{+}$, can be canceled in the region affected by the resonator. Therefore, the desired off-resonance frequency, $f_{0}$, should be above the Larmor frequency to enhance the transmit field. In general, transmit field efficiency is lower at the inferior region of the coil and higher compensation may be required. We adjust off-resonance frequency 5% above the Larmor frequency to obtain optimized transmit efficiency in the presence of the resonator [30,41]. The coupling between the resonator and the birdcage coil depends on the resonator orientation relative to the coil. Therefore, the transmit field profile of the resonator, $B_{re}$, depends on its relative orientation to the coil.

Inductive coupling of a matrix of resonators with the birdcage coil is more complicated than a coupling of a single resonator. All resonators in the matrix are inductively coupled to the MRI coil; therefore, their interaction is considered well in global homogenization. To this end, we performed full-wave electromagnetic simulations for more complementary results.

### 2.2. RF Enhancer

**Figure 2:**
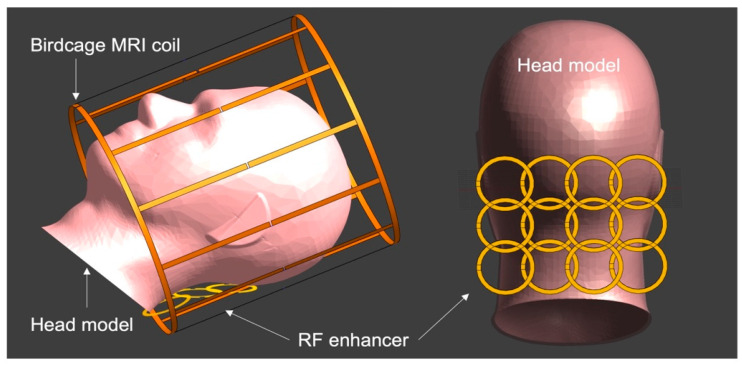
Schematic of the MRI birdcage head coil, RF enhancer, and head model. The enhancer is positioned at the base of the skull covering the posterior fossa (**right**). The head together with the enhancer goes inside the head coil (**left**).

The electrical characteristics of a single BC-SRR were analyzed with finite element simulations conducted in Sim4Life (Zurich Med Tech, Zurich, Switzerland) to optimize the design parameters including diameter, metallization width, and dielectric (interlayer) thickness. For the target operating frequency at 7T MRI (312 MHz, 5% above the Larmor frequency), a circular BC-SRR with a diameter of 60 mm, metallization width of 4 mm, and interlayer thickness of 250 μm was designed. An RF enhancer matrix consisting of 12 BC-SSRs (a 3 × 4 matrix) was created, with adjustment of distance between neighboring elements based on geometrical decoupling technique (0.76d mm, center-to-center) to minimize the coupling between the elements (Figure 2). We evaluated the effect of the RF enhancer on the EM field and SAR distributions of the head coil within the head model (relative permittivity of 45 and conductivity of 0.87 S/m) while the enhancer was placed between the head and the coil (Figure 2).

The birdcage head coil was designed similar to the transmit head coil (Nova Medical, Wilmington, MA, USA) used in the MRI experiments. The coil had 12 rungs connected at each end to two end rings and was shielded by an open cylinder (23 cm in diameter and 27 cm in length). Any EM interference may impact the coil electrical characteristics; therefore, the effect of inserting the wireless RF enhancer inside the coil was assessed by analyzing the S-parameters through EM simulations. A single BC-SRR was prototyped using the optimized design parameters obtained from EM modeling (mentioned above). For BC-SRR fabrication, a copper layer of a circular resonator ring was patterned on one side of a flexible interlayer substrate (Kapton, polyimide films, DuPont™, San Diego, CA, USA). Subsequently, another copper layer of a circular resonator ring was patterned on the opposite side of the substrate with a counter orientation, but aligned along the same axis as the first layer. The distributed capacitance between two conductor layers was employed to finely tune the frequency. Adjusting the gap size can affect both capacitance and inductance values, thereby impacting the overall operating frequency.

The benchtop resonance behavior was studied by measuring the reflection coefficient (S_11_) using a calibrated vector network analyzer (VNA, E5071C, Agilent Technologies, Santa Clara, CA, USA) that was directly connected to a single sniffer probe (a simple small loop coil). The decoupling level between the elements was evaluated by measuring transmission coefficient (S_21_) using coupled double pick-up probe technique. The double pick-up probe technique uses two overlapped small (1.5 cm in diameter) sniffer coils made from semi-rigid coaxial cable. During the benchtop experiment, the sniffer loop 1 that is connected to port 1 of the VNA transmits RF energy to the resonant element under test and sniffer loop 2 that is connected to port 2 of the VNA as a pick-up coil to detect currents circulated in the resonator under test. The quality factor (Q-factor) of each BC-SRR was calculated as $f_{0}$/Δf, where Δf is the FWHM bandwidth of the measured S_21_ using the double pick-up probe. Benchtop experiments were conducted under loaded and unloaded (free space) conditions. For loaded conditions, we used a cylindrical phantom (15 cm in diameter and 30 cm in height; relative permittivity: 49; conductivity: 0.81 s/m).

The distributed capacitance between two metal layers is one of the key factors that controls the operating frequency and is governed by the interlayer thickness. The distributed capacitance is inversely proportional to the interlayer thickness. A thinner interlayer confines higher electric field intensities in the interlayer region to achieve higher distributed capacitance, resulting in lowered $f_{0}$. The effect of the interlayer thickness was evaluated by measuring S-parameters of BC-SRRs with different interlayer thicknesses. We also assessed the bending effect on the $f_{0}$ under various bending conditions.

### 2.3. MRI Experiments

We conducted the phantom experiments to examine the voxel-wise behavior of the RF enhancer on $B_{1}^{+}$ efficiency and signal sensitivity ($B_{1}^{-}$). Measurements of $B_{1}^{+}$ and $B_{1}^{-}$ values at varying distances from the enhancer were taken under different applied RF voltages. In vivo MRI experiments were conducted on three healthy subjects on a 7T scanner (Magnetom, Siemens Healthineers, Erlangen, Germany) using single-channel transmit and 32-channel receive (1Tx/32Rx) Nova head coil. The human experimental procedures were approved by the local institutional review board. During the in vivo experiment, the enhancer was seated within the inferior position of the coil behind the upper (suboccipital) neck and covered the posterior fossa to recover the signal dropout due to a lower $B_{1}^{+}$ efficiency in this region.

In vivo dMRI data were obtained with and without the enhancer with 1.05 mm isotropic resolutions, GRAPPA acceleration factor = 3, repetition time/echo time (TE/TR) = 67.6/7200 ms, b-value = 1500 s/mm^2^, field-of-view (FOV) = 210 × 210 mm^2^, multiband acceleration factor = 2, total scan time = 18.5 min, 64 diffusion directions, and four b0 acquisitions, each acquired twice with anterior–posterior (AP) and PA phase encode directions to allow gradient nonlinearity correction in post-processing.

In vivo dMRI data were first evaluated by calculating the SNR on a voxel-wise basis in the cerebellum from b0 images acquired with and without the enhancer in place. The diffusion weighted imaging (DWI) series from both AP and PA directions were then compiled into a single volume, and the combined diffusion image was denoised and corrected for eddy-current distortions, subject motion, and $B_{1}^{+}$ inhomogeneity using MRtrix [42]. The post-processed diffusion data were subsequently used to generate whole-brain tractography using TractSeg [43], of which tracts of the posterior fossa were selected for study. These tracts are the corticospinal tract (CST), superior cerebellar peduncle (SCP), and inferior cerebellar peduncle (ICP).

## 3. Results

The coupling between the applied magnetic field and BC-SRR induces a surface current on the metallization layers, resulting in an additional H-field near the resonator (Figure 1). The E-field at the dielectric layer between conductive metallization layers indicates that the E-field is mainly confined in the interlayer region and does not spill over to the surrounding tissue.

**Figure 3:**
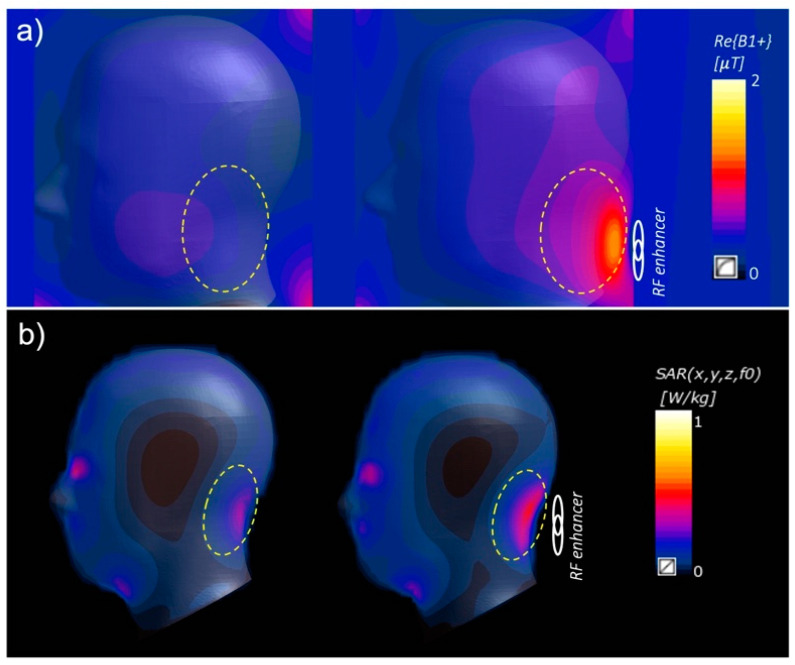
(**a**) The use of the enhancer resulted in a 2.1-fold improvement in transmit efficiency in the ROI (encircled with dashed yellow) in the presence of the RF enhancer (left) compared to the cases without the enhancer (right). (**b**) In the presence of the RF enhancer, the local SAR increased by 38% in the area covered by the enhancer, as indicated by a circle.

Similar to a single resonator, the interaction of the RF enhancer (matrix of BC-SRRs) with the EM field generated by the MRI coil caused a secondary magnetic field near the enhancer, leading to an improvement in the total magnetic field near the enhancer. Numerical simulations show that the presence of the enhancer resulted in 2.1-fold enhancement in $B_{1}^{+}$ efficiency in the region of interest (ROI) encircled with dashed yellow lines (Figure 3a). SAR simulations with 10 gr averaging were studied to assess the presence of the RF enhancer in the SAR distribution in the head model. These results show that by placing the enhancer, the SAR level is increased 38% 10 gr of averaging in the ROI encircled with dashed yellow (Figure 3b). These numbers should be considered to determine the upper limit of the applied RF power for the imaging sequences to stay within the safe levels.

**Figure 4:**
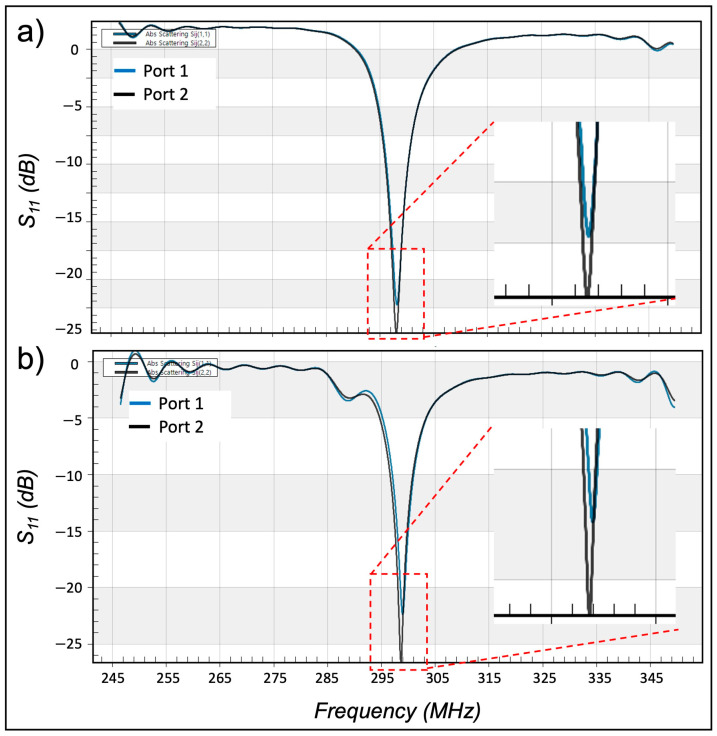
Effect of the RF enhancer on the resonance behavior of the birdcage coil. Comparing the S_11_ results from the coil without the enhancer (**a**) and the coil with the enhancer (**b**) shows that adding the enhancer results in a slight up-shift (>1 MHz) in the resonance frequency of the birdcage coil.

Comparing the simulation results without (Figure 4a) and with (Figure 4b) the RF enhancer also show that inserting the RF enhancer inside the coil causes a slight shift (<1 MHz) in the resonance behavior of the coil.

**Figure 5:**
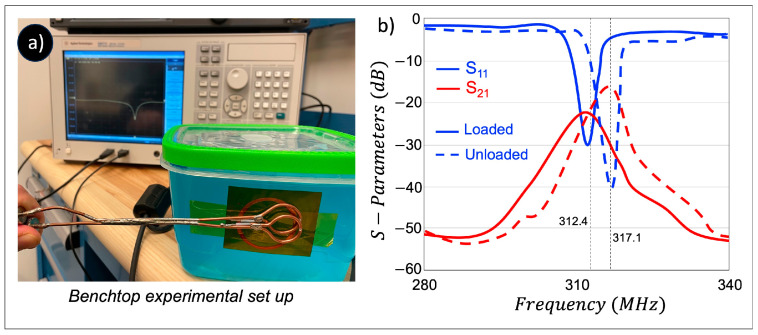
(**a**) Benchtop test setup for measuring the resonance behavior of a single BC-SRR. (**b**) S-parameter measurement results for a BC-SRR under test in both unloaded (dashed line) and loaded (solid line) conditions.

**Figure 6:**
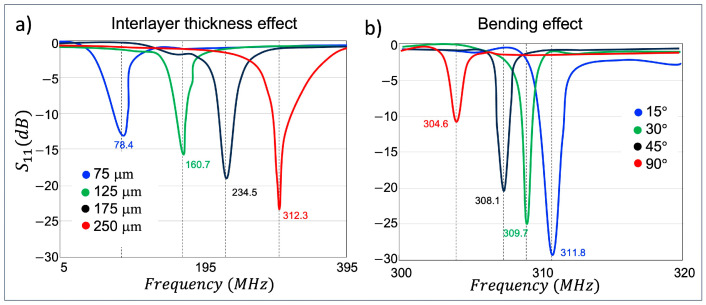
Evaluation of the effect of the interlayer thickness (**a**) and bending effect (**b**) on the resonance behavior of a single BC-SRR.

The BC-SRR electrical characteristics are studied in terms of the measured S-parameters and Q-factor using a VNA under unloaded and loaded conditions (Figure 5a). The S-parameter measurements of a single BC-SRR are shown in Figure 5b. Although there is a slight difference in the dielectric properties, the variations are minimal and are not expected to significantly impact the comparability of the simulation and experimental results. An average loaded Q-factor of 14 and an average unloaded Q-factor of 31 were calculated. We evaluated the interlayer thickness effect on the $f_{0}$ under various thicknesses of 70 μm, 125 μm, 175 μm, and 250 μm (Figure 6a). Thinner interlayers led to higher distributed capacitance, consequently resulting in lowered $f_{0}$. We also assessed the bending effect on the $f_{0}$ under various bending conditions of 15°, 30°, 45°, and 90° which caused a 0.5 MHz, 1.1 MHz, and 1.8 MHz up-shift in the frequency, respectively (Figure 6b).

BC-SRRs with optimized design parameters, tuned to 312 MHz (5% above the Larmor frequency at 7T), were used to build a matrix of resonators. The resonator elements are decoupled from each other using the critically overlapping technique. From the perspective of S-parameter measurement, to minimize the inductive decoupling between resonators, the distance between neighboring resonators should be adjusted such that the frequency with a minimum S_21_ is equal to the tuned frequency, 312 MHz. Decoupling between two resonators was assessed by measuring S_21_ while all other neighboring resonators were detuned. The coupling measurements of a single resonator relative to the adjacent resonators show an acceptable decoupling level between the neighboring resonators (<−17 dB).

Specific safety considerations are necessary to assess the interaction of the applied EM field with the surface array so that the SAR level and tissue heating are not significantly elevated. Following a suitable modification of the ASTM standards (Supplementary Materials), a safety heating test was performed to ensure that tissue heating remained below established safety limits [44,45].

**Figure 7:**
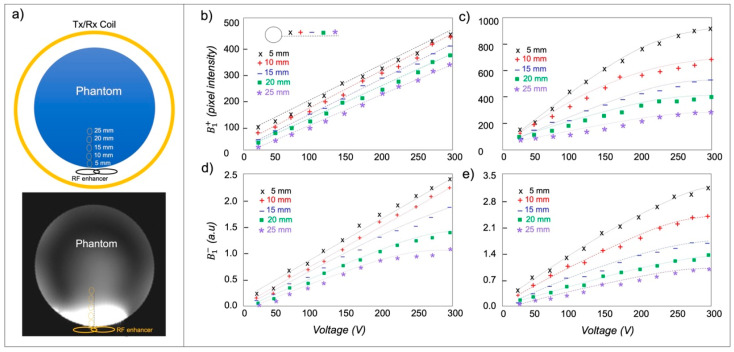
(**a**) Schematic of the MRI phantom experiment with the corresponding MRI images in the coronal plane with the enhancer. (**b**) Pixel-wise $B_{1}^{+}$ intensity from different distances under varying applied RF voltage without using the enhancer. (**c**) Pixel-wise $B_{1}^{+}$ intensity from different distances under varying applied RF voltage using the enhancer. (**d**) $B_{1}^{-}$ changes by varying the applied RF voltage at different distances without the enhancer. (**e**) $B_{1}^{-}$ changes by varying the applied RF voltage at different distances in the presence of the enhancer.

For the phantom MRI experiment, the RF enhancer was positioned inside the head coil, particularly in regions with lower transmit efficiency, with the phantom placed on top of the enhancer (Figure 7a). The experiment was conducted both with and without the enhancer in place under different applied voltages. When the enhancer was absent, the $B_{1}^{+}$ value exhibited a linear increase with the applied RF voltage at different distances from the enhancer (Figure 7b). With the enhancer in place, the $B_{1}^{+}$ value increased linearly with the applied RF voltage up to approximately 200 V (Figure 7c), beyond which the rate of increase in $B_{1}^{+}$ value decreased due to the RF over-flipping. On average, the addition of the enhancer resulted in a $B_{1}^{+}$ enhancement of 2.3-fold across all applied voltages and distances. Assessing the $B_{1}^{-}$ sensitivity without the enhancer revealed that increasing the applied voltage led to an increase in the $B_{1}^{-}$ value (Figure 7d). However, at greater distances from the enhancer, the rate of increase in $B_{1}^{-}$ decreased at higher voltage levels due to approaching the Ernst angle. In the presence of the enhancer, increasing the applied voltage resulted in an increase in $B_{1}^{-}$ values, but at higher voltages, the rate of increase decreased (Figure 7e). Comparing $B_{1}^{-}$ values, the presence of the enhancer yielded an average enhancement of 1.7-fold.

The spatial distribution of the $B_{1}^{+}$ maps in the in vivo MRI experiments showed that presence of the enhancer improved performance in the posterior fossa. The average transmit efficiency across the ROI showed a mean 1.9-fold improvement in the presence of the enhancer. This improvement may transfer to the SNR enhancement in the MR images. Quantitatively comparing $B_{1}^{-}$ maps obtained without the enhancer and using the enhancer showed an average of 1.4-fold enhancement in the ROI in the presence of the enhancer. A qualitative assessment exhibited that the application of the enhancer effectively improved the MR signal and recovered the signal dropouts within the region (See Supplementary B for detailed explanations).

**Figure 8:**
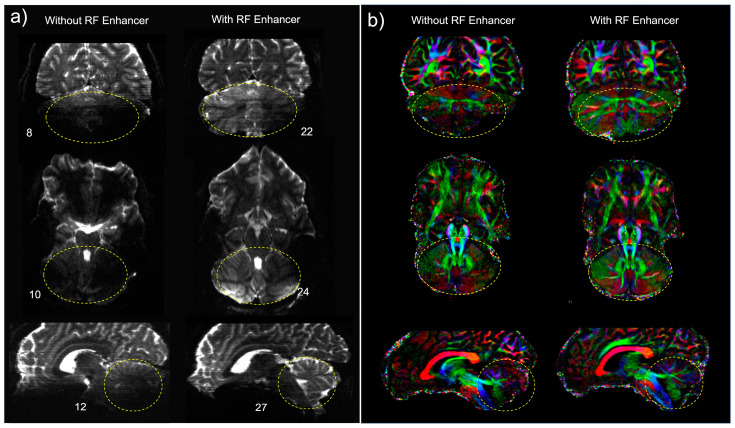
(**a**) Comparison of b0 images of dMRI data acquisition using the single-channel transmit coil without the enhancer (left column) and with the enhancer (right column). Note that the use of the RF enhancer increased the temporal SNR across the cerebellum and brain stem (encircled by dashed yellow) by an average factor of 2.4. Numbers indicate the temporal SNR in the ROI. (**b**) Comparing color fractional anisotropy (FA) maps (in the range of [01]) obtained using a single channel transmit head coil without the enhancer (left column) and with the enhancer (right column). The color representing the orientation of the principal fiber (red: left-right; green: A-P; blue: inferior-superior). The result was a clearer depiction of fibers and better estimation of multiple fiber orientations.

The analysis of dMRI with and without the enhancer indicated improved SNR and contrast in the posterior fossa in the b0 images in three different planes (Figure 8a). The temporal SNR (tSNR) was calculated by dividing the mean signal intensity over time by the standard deviation of the pixel noise over the time course. The standard deviation of the noise was measured once the RF voltage was set up to zero. The tSNR enhancement of 2.7-fold, 2.4-fold, and 2.2-fold was calculated in the coronal, axial, and sagittal planes, respectively. tSNR maps with and without the RF enhancer are shown in Supplementary Figure S4. To isolate the effect of the enhancer, FA maps were obtained in three different planes to compare fibers conspicuity in the presence of the RF enhancer. The use of the standard 1Tx head coil without the enhancer yielded lower signal intensity in the cerebellum. This translated into noisy depictions of principle fiber orientations within these regions. In contrast, the use of the enhancer significantly improved local signal sensitivity, and thereby the SNR. The use of the enhancer recovered tissue in the cerebellum and improved fiber orientation estimation performances in this region (Figure 8b).

**Figure 9:**
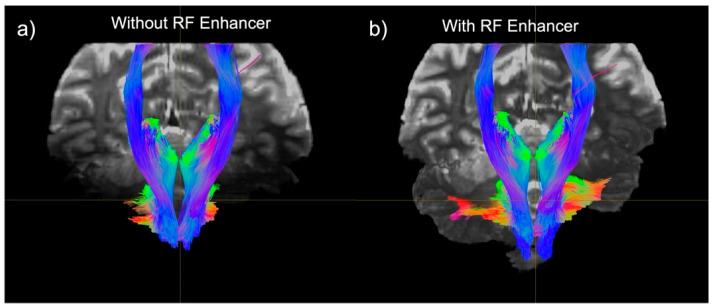
Comparison of tractography generated using a 1Tx head coil without the enhancer (**a**) and with the enhancer (**b**). The fibers shown make up the primary white matter pathways of the brainstem and cerebellum: the corticospinal tract, and the superior and inferior cerebellar peduncles. Tracts are overlaid onto their respective b0 images.

We subsequently generated tractography maps to analyze the effect of the RF enhancer on white matter pathway visualization. We found an improvement in the SNR resulting in the enhanced visualization of tracts in the cerebellum and brainstem. The improvement in the SNR can be seen as an increased conspicuity in the cerebellar and corticospinal tracts, and in the increased coverage of the cerebellum in the background b0 image (Figure 9).

## 4. Discussion

In this study, we demonstrated how a novel inductively coupled RF enhancer improves dMRI at 7T. The wireless RF enhancer is a thin and flexible structure consisting of multiple BC-SRRs tuned to operate at 7T MRI. The proposed device works based on the inductive coupling between the MRI coil and the enhancer during both the RF transmission and signal reception. The in vivo placement of the enhancer inside the head coil over the posterior fossa (Figure 2), a region with intrinsically reduced transmit efficiency, enhances $B_{1}^{+}$ efficiency to improve signal sensitivity. With this position, it was possible to closely examine the effectiveness of the surface enhancer on the human brain dMRI at 7T. We started by using EM simulation to optimize the design parameters for tuning the resonator’s operating frequency. Our prior publications have shown that the optimization of transmit efficiency enhancement with the RF enhancer requires the resonance frequency to be adjusted 5% above the Larmor frequency [30,41].

The placing of the RF enhancer in the caudal part of the head may cause bending on the device, which may affect the electrical characteristics and EM distribution. Through benchtop experiments, we demonstrated that the resonance frequency shift associated with varying bending angles was negligible. Research by other groups indicated that bending the resonator up to 45° did not significantly affect the field distributions [23,46].

The mechanism through which the enhancer increases SNR lies in $B_{1}^{+}$ efficiency and $B_{1}^{-}$ sensitivity within the area affected by the enhancer. The enhancer yields more uniform magnetic field distribution across the anatomy of interest. However, the enhancement of the electric field may result in an elevated SAR, raising safety concerns related to tissue heating. Heating test based on the ASTM guideline reported a 34% increase in the SAR. Therefore, to safely conduct the scan in the presence of the enhancer, the SAR limits applied by the sequence should not exceed 66%.

The in vivo performance of the enhancer was assessed in three human subjects. The integration of the enhancer with the head coil led to a 2.1-fold increase in $B_{1}^{+}$ efficiency and a 1.4-fold increase in normalized $B_{1}^{-}$ sensitivity enhancement within the ROI. These enhancements resulted in a clear structural visualization and better depiction of white matter bundles and fiber orientation.

We have designed this device to recover the signal dropouts in the regions with an intrinsically lower transmit efficiency without material degradation or complex hardware requirements. This proposed technique to improve dMRI at 7T can be used in conjunction with other $B_{1}^{+}$ shimming methods such as dielectric pads and pTx systems. Studies by our team and others showed that a hybrid configuration through combining a resonators structure with a high permittivity material might improve the MRI at 7T [23,28]. Combining the RF enhancer with pTx systems may require additional SAR safety evaluations due to the interaction of the varying profile of the EM filed with the device, which may cause high SAR hot spots. We also compared the performance of the RF enhancer with that of the dielectric pads. T2-weighted TSE images obtained with both the dielectric pads and the RF enhancer demonstrated improvements in the MRI signal. However, the RF enhancer provided a higher SNR enhancement, resulting in better anatomical coverage (See Supplementary Figure S5). It is important to note that strong coupling between the coil and the enhancer may cause RF over-flipping and non-uniform signal enhancement due to flip angle inhomogeneity. To prevent over-flipping, we used antiparallel cross-diodes in the elements positioned in areas of strong coupling. Another possible solution is to adjust the distance between the head and the enhancer.

## 5. Conclusions

The present study evaluated the advantages of using the RF enhancer in conjunction with 1Tx head coil configurations for improving cerebellar dMRI at 7T. The results show that using the device can significantly improve $B_{1}^{+}$ efficiency and signal sensitivity in dMRI of the posterior fossa compared with a standard 1Tx configuration. Most notably, the use of the enhancer allowed for a better depiction of principal fiber orientations and an enhanced visualization of the extended tracts to the brainstem and pons, enabling a more comprehensive delineation of the full white matter architecture. In conclusion, there is potential for the proposed enhancer to facilitate the rapid acquisition of high-quality, high-resolution, whole-brain dMRI, which is desirable for many neurological and clinical applications. Further studies are underway to incorporate the enhancer into standard clinical and research indications in order to determine its practical utility within a 7T workflow. The clinical importance of accurately mapping white matter tracts is particularly salient when considering individualized treatment approaches for conditions such as epilepsy and movement disorders, where neurosurgical interventions with stimulation are increasingly common. The conceptualization of such pathologies as network disorders emphasizes the importance of delineating precise structural connectivity, particularly involving the brainstem, to inform surgical planning and diagnostics. The next step will involve developing a larger version of the RF enhancer to cover a broader region, specifically targeting the temporal lobe, where the wavelength effect is more pronounced. One limitation of this study is the use of a homogeneous head phantom; multilayer phantoms could be employed in future work to more accurately represent the complexities of human tissue. Multilayer phantoms are particularly useful in electromagnetic MRI simulations as they can mimic the varying electrical properties of different tissue layers, leading to more precise modeling of interactions between electromagnetic fields and biological tissues.

## Data Availability

Data are contained within the article and Supplementary Materials.

## Supplementary Materials

The following supporting information can be downloaded at https://www.mdpi.com/article/10.3390/s24216981/s1.
