# Supplementary material for: Radiofrequency Enhancer to Recover Signal Dropouts in 7 Tesla Diffusion MRI
Source: Sensors (Basel). 2024 Oct 30;24(21):6981. doi: 10.3390/s24216981 (PMC11548241; doi:10.3390/s24216981)
Supplement: Supplementary file 1 [file sensors-24-06981-s001.zip › sensors-3144295-supplementary.pdf]

Supplementary Document

Radiofrequency Enhancer to Recover Signal Dropouts in 7 Tesla Diffusion MRI

Structure

Varun Subramaniam[1], Andrew Frankini[1], Ameen Al Qadi[1], Mackenzie T. Langan[1], Gaurav Verma[1], Bradley N Delman[1], Priti Balchandani[1,2], and Akbar Alipour[1,*]

Supplementary A: Equation derivations

Assume the CP magnetic field generated by the MRI coil (quadrature birdcage) expressed as

$$\boldsymbol{B}_{rf}(t) = B_1^+(cos\ \omega_L t\ \boldsymbol{i} - sin\ \omega_L t\ \boldsymbol{j})$$

$$\boldsymbol{B}_{re}(t) = 2B_{re}^+ \cos \omega_L t\ \boldsymbol{i}$$

Assume the angle between CP magnetic field lines and normal vector of the resonator is zero, therefore, from Faraday's law of induction, the electromotive force (ε) generated by $\boldsymbol{B}_{rf}(t)$ is given:

$$\epsilon = -\frac{d\phi}{dt} = (\pi r^2)\omega_L B_1^+$$

If the resonator is considered as a series RLC circuit, the input impedance can be written as:

$$Z_{re} = R\left[1 + i\frac{\omega_L L}{R}\left(\frac{f_L^2 - f_0^2}{f_L^2}\right)\right] = R\left[1 - i\frac{\omega_L L}{R}\left(\frac{(f_0 - f_L)(f_0 + f_L)}{f_L^2}\right)\right]$$

Assuming $\Delta f = f_0 - f_L$ and $f_0 \cong f_L$, using Taylor expansion,

$$Z_{re} = R\left[1 - i\frac{\omega_L L}{R}\left(\frac{2\Delta f f_L}{f_L^2}\right)\right] = R\left[1 - i2\frac{\omega_L L}{R}\left(\frac{\Delta f}{f_L}\right)\right]$$

The ohmic loss is typically small and the impedance can be approximated as:

$$Z_{re} = -i2\omega_L L\left(\frac{\Delta f}{f_L}\right)$$

The associated ohmic loss, R, is typically small, therefore the induced current on the resonator can be written as:

$$I_{re} = \frac{\epsilon}{Z_{re}} \simeq \frac{(\pi r^2)\omega_L B_1^+}{i2\omega_L L\left(\frac{\Delta f}{f_L}\right)} = \frac{(\pi r^2)B_1^+}{2L}\left(\frac{f_L}{\Delta f}\right)$$

The modulation magnetic field generated by the induced current at distance z away from the resonator center is given by:

$$\boldsymbol{B}_{re}(t) \simeq \frac{\mu\,(\pi r) B_1^+}{L\left(1+\left(\frac{z}{r}\right)^2\right)^{\frac{3}{2}}}\left(\frac{f_L}{\Delta f}\right) cos\ \omega_L t\ \boldsymbol{i}$$

$$\boldsymbol{B}_{re}(t) \simeq \frac{\mu\,(\pi r) B_1^+}{2L\left(1+\left(\frac{z}{r}\right)^2\right)^{\frac{3}{2}}}\left(\frac{f_L}{\Delta f}\right)[cos\ \omega_L t\ \boldsymbol{i} - sin\ \omega_L t\ \boldsymbol{j}] + \frac{\mu\,(\pi r) B_1^+}{2L\left(1+\left(\frac{z}{r}\right)^2\right)^{\frac{3}{2}}}\left(\frac{f_L}{\Delta f}\right)[cos\ \omega_L t\ \boldsymbol{i} + sin\ \omega_L t\ \boldsymbol{j}]$$

$$\begin{aligned}\boldsymbol{B}_{\boldsymbol{t}}^+(t) &\simeq \boldsymbol{B}_{rf}(t) + \frac{\mu\pi\, B_1^+}{2L\left(1+\left(\frac{z}{r}\right)^2\right)^{\frac{3}{2}}}\left(\frac{f_L}{\Delta f}\right)[cos\ \omega_L t\ \boldsymbol{i} - sin\ \omega_L t\ \boldsymbol{j}] \\ &= \left[1 + \frac{\mu\,\pi}{2L\left(1+\left(\frac{z}{r}\right)^2\right)^{\frac{3}{2}}}\left(\frac{f_L}{\Delta f}\right)\right] B_1^+[cos\ \omega_L t\ \boldsymbol{i} -\ sin\ \omega_L t\ \boldsymbol{j}]\end{aligned}$$

Supplementary B: Heating test

Inductive coupling between the RF enhancer and MRI coil may boost the electric field and cause tissue heating. The heating test was performed to access the RF-induced heating on or near the wireless array and its surroundings during RF transmission. The test was evaluated by suitable modification of the method described in the ASTM F2182 standard [1]. To assess the effect of the enhancer in the absorbed RF energy, the SAR was calculated experimentally, $SAR = C_{gel}\,(dT/dt)$ using the heating test, based on ASTM guideline in a gel phantom (Permittivity = 77, conductivity = 0.50 S/m, heat capacity (C_{gel}) = 4154 J/kg°C) prior to in-vivo experiments, where T is the temperature and t is the time. Five fiber optic temperature probes (LumaSense Technologies, Santa Clara, California, USA) were placed in the phantom close to the enhancer at locations predicted using SAR simulations to experience high SAR. The array was isolated using a thin layer of polymer to avoid the direct contact of the array with the gel material. These locations included the gap edges and capacitive regions of the elements, which were in a strong coupling position. Figure S1 shows the heating test setup. A high SAR turbo-spin-echo sequence (TSE, TR/TE = 500/11 ms, flip angle (FA) = 180°, average = 32, matrix = 256 × 256) with a high RF duty cycle was run for 15 minutes. Baseline temperatures were recorded before RF transmission for 2 min, and temperature changes were

measured during scanning. Afterward, the array was removed, and after returning the gel temperature to the thermal equilibrium, the test was repeated with the temperature measurement probes placed at the exact locations. In all measurements (with/without the array), the position of the fiberoptic probes relative to the phantom was kept constant. The SAR gain determining the safety factor was calculated from the temperature measurements of each probe location. Figure 2S of the supplementary document depicts the details of the setup. Electromagnetic (EM) SAR simulations were performed to evaluate the SAR distribution near the resonator and the wireless array

We performed SAR simulations with the RF enhancer (2-element) to detect the possible high SAR hot spots for heating test (Figure S2).

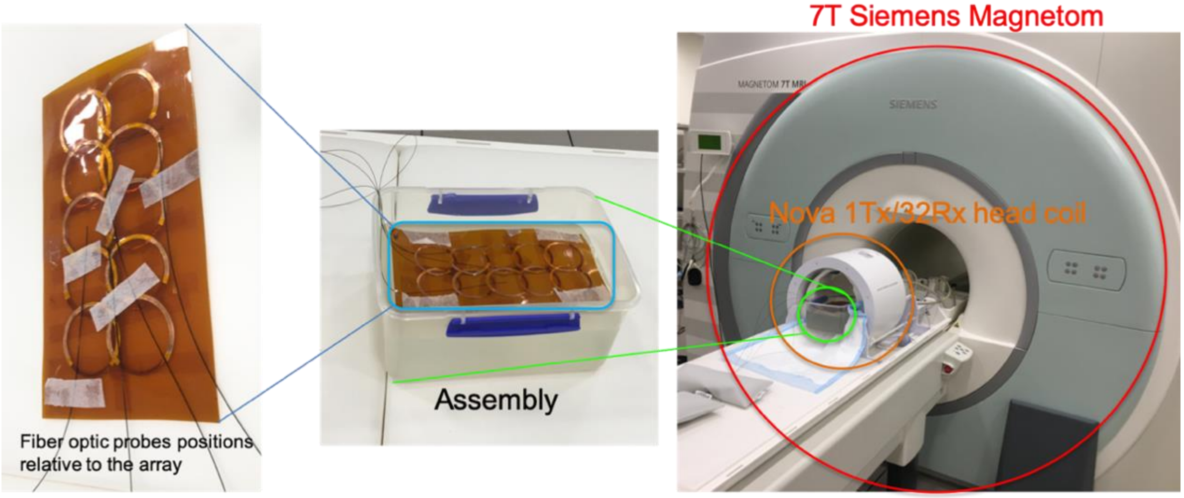

Figure S1. Set up for temperature measurements at 7T MRI using gel phantom. Please note that during the experiment the wireless array was placed on the bottom of the gel container.

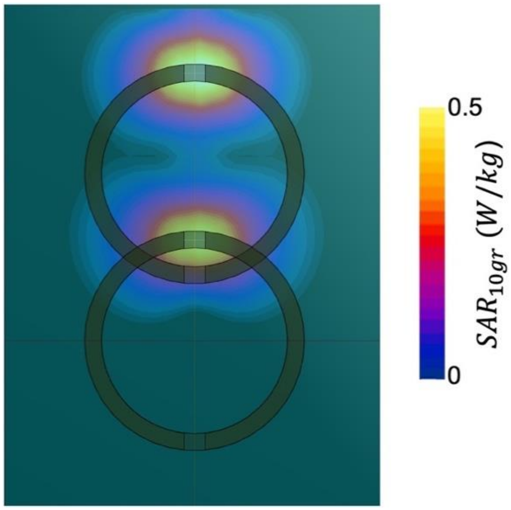

Figure S2. 10 gr average SAR distribution of two geometrically decoupled resonators. High SAR hot spots are located on the gap regions due to high electric field confinement in those regions.

After 15 minutes of RF excitation, a maximum temperature increases of about 0.71°C was reported in the vicinity of the RF enhancer, while the temperature rise recorded by the reference probe was 0.5 °C. Considering the associated SAR gain of 1.42, the SAR limits should not exceed 58% when the RF array is in place to safely perform scanning. These numbers should be considered to determine the upper limit of the applied RF power for the imaging sequences for the patients with such an array. Based on the test results, the coil file can be modified to limit the SAR level. Another approach is monitoring the SAR level on the MRI workstation to ensure it does not exceed the modified limit.

Supplementary C: B_1^+ and B_1^- mapping

To assess the in-vivo performance of the RF enhancer, B_1^+ and B_1^- maps were calculated with/without the enhancer in place. Transmit RF efficiency was evaluated by mapping the B_1^+ produced in the phantom using the presaturation-prepared turbo-FLASH based method with acquisition parameters TR/TE=2.7/1.2 ms, flip angel=10°, field-of-view (FOV)= 16 cm×16 cm, matrix= 256×256. This condition was used to measure the transmitter amplitude for each pixel [2]. B_1^- maps were generated using the images obtained from two gradient-recalled echo sequences (GRE, TR/TE= 400/9 ms, flip angle= 10°, bandwidth= 977 Hz/pixel, FOV= 16 cm×16 cm, matrix= 256×256), one with and the other without RF transmission and normalized by the sine of the corresponding flip angle map to isolate the receive sensitivity from the transmit field distribution [3]. The input power was kept the same either with and without the surface array modes in B_1^+ and B_1^- maps calculations. To qualitatively assess the effect of adding the enhancer, we also acquired images using turbo spin echo (TSE) sequences (TR/TE = 3000/76 ms, FOV = 22 cm×18 cm, flip angle = 120°, slice thickness = 2 mm, bandwidth = 977 Hz/pixel, matrix = 256×256, TSE factor = 15.

The spatial distribution of the B_1^+ maps in the in-vivo MRI experiments showed that present of the enhancer results in 1.9-fold enhancement in the ROI (Figure S3a). Quantitatively comparing B_1^- maps obtained without the enhancer and using the enhancer showed an average of 1.4-fold enhancement in the ROI in the presence of the enhancer (Figure S3b).

The temporal SNR (tSNR) maps in the in-vivo MRI experiments also showed that present of the enhancer results in 2.2-fold enhancement in the ROI encircled with dashed yellow (Figure S4).

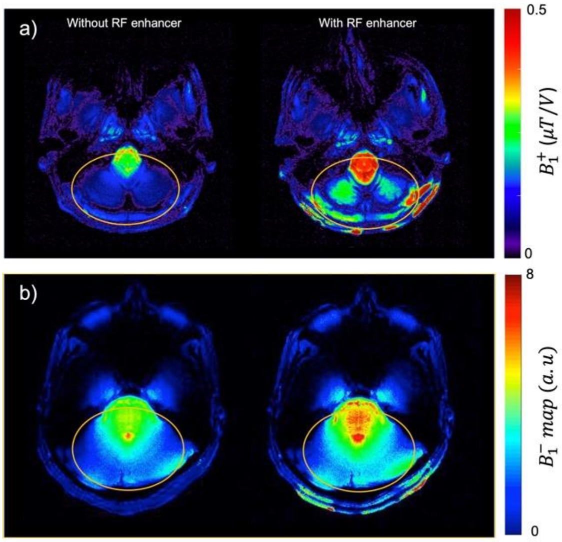

Figure S3. In-vivo B_1^+ efficiency maps without (a) and with (b) the enhancer shows an improvement with the RF enhancer in the inferior-posterior region of the brain. Average B_1^+ enhancement of a factor of 2$\pm$0.3 was calculated across the region of interest including the cerebellum and brainstem (encircled by orange). In-vivo signal sensitivity, B_1^- maps without (c) and with (d) the enhancer shows an enhancement with the RF enhancer in the same region of the brain. Average B_1^- enhancement of a factor of 0.4 was calculated across the interested region (encircled by orange).

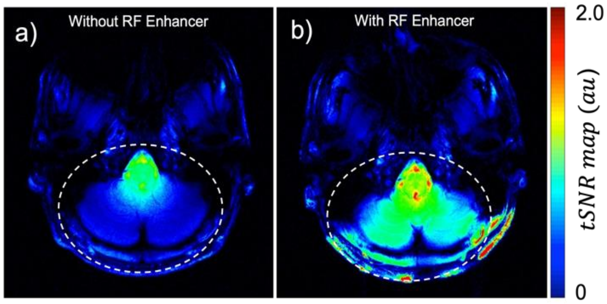

Figure S4. In-vivo tSNR maps without (a) and with (b) the enhancer shows an enhancement with the RF enhancer in the inferior-posterior region of the brain. The tSNR enhancement of a factor of 2.2 was calculated across the interested region (encircled by dashed yellow).

Supplementary D: Comparing the RF enhancer with the dielectric pad

We also compared the performance of the RF enhancer with that of the dielectric pads. T2-weighted TSE images obtained with both the dielectric pads and the RF enhancer demonstrated improvements in MRI signal. However, the RF enhancer provided a higher SNR enhancement, resulting in better anatomical coverage (Figure S5).

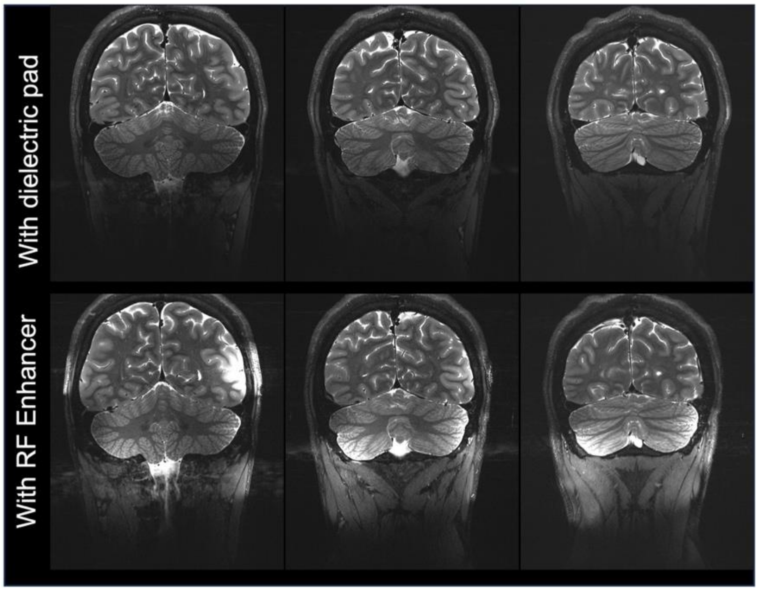

Figure S5. Comparison of T2-weighted TSE images obtained using dielectric pads (top row) and an RF enhancer (bottom row) at 7T MRI. Both techniques enhance the MRI signal, with qualitative analysis indicating that the RF enhancer provides a higher SNR.

References:

1. Standard Test Method for Measurement of Radio Frequency Induced Heating On or Near Passive Implants During Magnetic Resonance Imaging. F2482-11aASTM F2482-11a2011. doi:10.1520/F2182-11A.

2. Tournier, J.D., et al., MRtrix3: A fast, flexible and open software framework for medical image processing and visualisation. NeuroImage, 2019. 202: p. 116137.

3. Wasserthal, J., P. Neher, and K.H. Maier-Hein, TractSeg - Fast and accurate white matter tract segmentation. NeuroImage, 2018. 183: p. 239-253.
